# Electron Tomography Reveals the Steps in Filovirus Budding

**DOI:** 10.1371/journal.ppat.1000875

**Published:** 2010-04-29

**Authors:** Sonja Welsch, Larissa Kolesnikova, Verena Krähling, James D. Riches, Stephan Becker, John A. G. Briggs

**Affiliations:** 1 Structural and Computational Biology Unit, European Molecular Biology Laboratory, Heidelberg, Germany; 2 Institut für Virologie, Philipps-Universität Marburg, Marburg, Germany; Institut Pasteur, France

## Abstract

The filoviruses, Marburg and Ebola, are non-segmented negative-strand RNA viruses causing severe hemorrhagic fever with high mortality rates in humans and nonhuman primates. The sequence of events that leads to release of filovirus particles from cells is poorly understood. Two contrasting mechanisms have been proposed, one proceeding via a “submarine-like” budding with the helical nucleocapsid emerging parallel to the plasma membrane, and the other via perpendicular “rocket-like” protrusion. Here we have infected cells with Marburg virus under BSL-4 containment conditions, and reconstructed the sequence of steps in the budding process in three dimensions using electron tomography of plastic-embedded cells. We find that highly infectious filamentous particles are released at early stages in infection. Budding proceeds via lateral association of intracellular nucleocapsid along its whole length with the plasma membrane, followed by rapid envelopment initiated at one end of the nucleocapsid, leading to a protruding intermediate. Scission results in local membrane instability at the rear of the virus. After prolonged infection, increased vesiculation of the plasma membrane correlates with changes in shape and infectivity of released viruses. Our observations demonstrate a cellular determinant of virus shape. They reconcile the contrasting models of filovirus budding and allow us to describe the sequence of events taking place during budding and release of Marburg virus. We propose that this represents a general sequence of events also followed by other filamentous and rod-shaped viruses.

## Author Summary

The filoviruses, Marburg and Ebola, cause lethal hemorrhagic fever and are highest-priority bioterrorism agents. Filovirus particles contain a rod-like nucleocapsid and are normally filamentous, though other shapes are seen. It is poorly understood how such large filamentous particles are assembled and released from infected cells. Here we have studied Marburg virus production in infected cells using electron tomography. This technique allows virus particles to be visualized in three dimensions at different stages during assembly. We find that in early stages of virus production, highly infectious filamentous viruses are produced, whereas after prolonged infection poorly infectious spherical viruses are released. We also define the sequence of steps in filamentous virus release. The intracellular nucleocapsid first travels to the plasma membrane of the cell, where it binds laterally along its whole length. One end is then wrapped by the plasma membrane and wrapping proceeds rapidly until the virus protrudes vertically from the cell surface. The rear end of the virus particle then pinches off from the cell. We propose that other important filamentous and rod-shaped viruses also follow this series of steps of assembly and budding.

## Introduction

Marburg virus (MARV) and Ebola virus, the two genera in the family *Filoviridae*, cause fulminant hemorrhagic disease in humans and nonhuman primates, resulting in high mortality rates [1], [2], [3]. Outbreaks of MARV disease in sub-Saharan Africa underline the emerging potential of this virus, which is classified as a highest-priority bioterrorism agent by the Centre for Disease Control [4], [5], [6], [7], [8].

The filoviruses are members of the order *Mononegavirales* and contain a single-stranded negative-sense RNA genome, which is encapsidated by the nucleoprotein (NP). The MARV genome encodes seven structural proteins [9], [10]: the polymerase (L), VP35 and VP30 associate with NP to generate the helical nucleocapsid (NC) [11], [12], [13]. The viral glycoprotein (GP), which is inserted in the viral envelope, mediates cell entry [14], [15]. The major matrix protein VP40 plays a key role in virus assembly, and VP24, the second matrix protein, is suggested to support the template function of the NC [12], [16], [17], [18].

MARV infected cells develop viral inclusions in the perinuclear region [19], [20], [21]. These contain NC proteins and are most likely centres of NC assembly [22]. MARV particles bud from the plasma membrane (PM) of long filamentous cellular protrusions that contain parallel actin bundles and other markers of filopodia [23]. The released virus particle has a membrane envelope and contains an NC that is surrounded by the viral matrix protein VP40. It is unclear how NCs are transported from viral inclusions to the PM, whether they adopt their virion conformation before, during, or after, transport, or where NCs associate with VP40 that is not co-transported with NCs but is necessary for budding. Released MARV particles appear filamentous, hooked, six-shaped or round by electron microscopy (EM) [24] but their three-dimensional (3D) morphology is unclear. It is also unknown whether production of differently shaped viruses depends on different budding mechanisms and whether they differ in infectivity. Experiments to address these issues are complicated by the need to perform all infection experiments under BSL-4 containment conditions.

The processes of assembly, budding and release of spherical viruses have been extensively studied [25], [26], [27] and it is well established that spherical enveloped viruses are produced by budding away from the cytoplasm in a process that is related and topologically equivalent to the formation of small vesicles in multivesicular bodies [28], [29]. In contrast, the basic steps of assembly and release for large, filamentous, enveloped particles such as the filoviruses are poorly understood. EM studies have shown MARV and Ebola virus particles protruding perpendicularly from the cell [23], [30]. These observations, together with similar findings of protruding intermediates of other important filamentous or rod-shaped viruses such as rabies virus [31], influenza virus [32], [33], [34], or vesicular stomatitis virus (VSV) [35] have led to the suggestion of a vertical “rocket-like” mode of budding. Ebola virus NCs have been seen associated parallel to the PM [36], leading to the suggestion that a second, horizontal, “submarine-like” mechanism is the major mode of budding. This unusual mechanism has not been described for any other virus.

In this study we use electron tomography (ET) to describe and analyse in 3D the structure of MARV budding intermediates and released viruses at different stages of infection. In contrast to conventional EM of ultrathin sections, ET allows complete MARV virions and budding structures to be studied in 3D. This permits unambiguous determination of virus morphology, dimensions, stage of budding and position relative to the infected cell. The samples are prepared by high-pressure freezing followed by embedding in resin and staining with heavy metals. Unlike cryo-ET of vitreous samples, this method is not appropriate for the study of high-resolution protein structure. Nevertheless, it gives excellent preservation of the features studied here such as membranes and protein assemblies including viral NCs [37], [38]. It also has, for this particular purpose, substantial advantages over cryo-ET. The features of interest are imaged at much higher contrast, including at high tilt angles. The samples are also easier to handle and stable under the electron beam, allowing efficient screening, which facilitates the collection of larger datasets.

Our observations suggest that interplay between the virus and the infection state of the cell determines virus morphology. Furthermore, the 3D data allow us to describe the sequence of steps that take place in infected cells during assembly and budding of filamentous virions, and to reconcile the horizontal and vertical models for filovirus budding as representing different snapshots of a single budding process.

## Results

### Characterisation of the course of MARV infection in cultured cells

**Figure 1:**
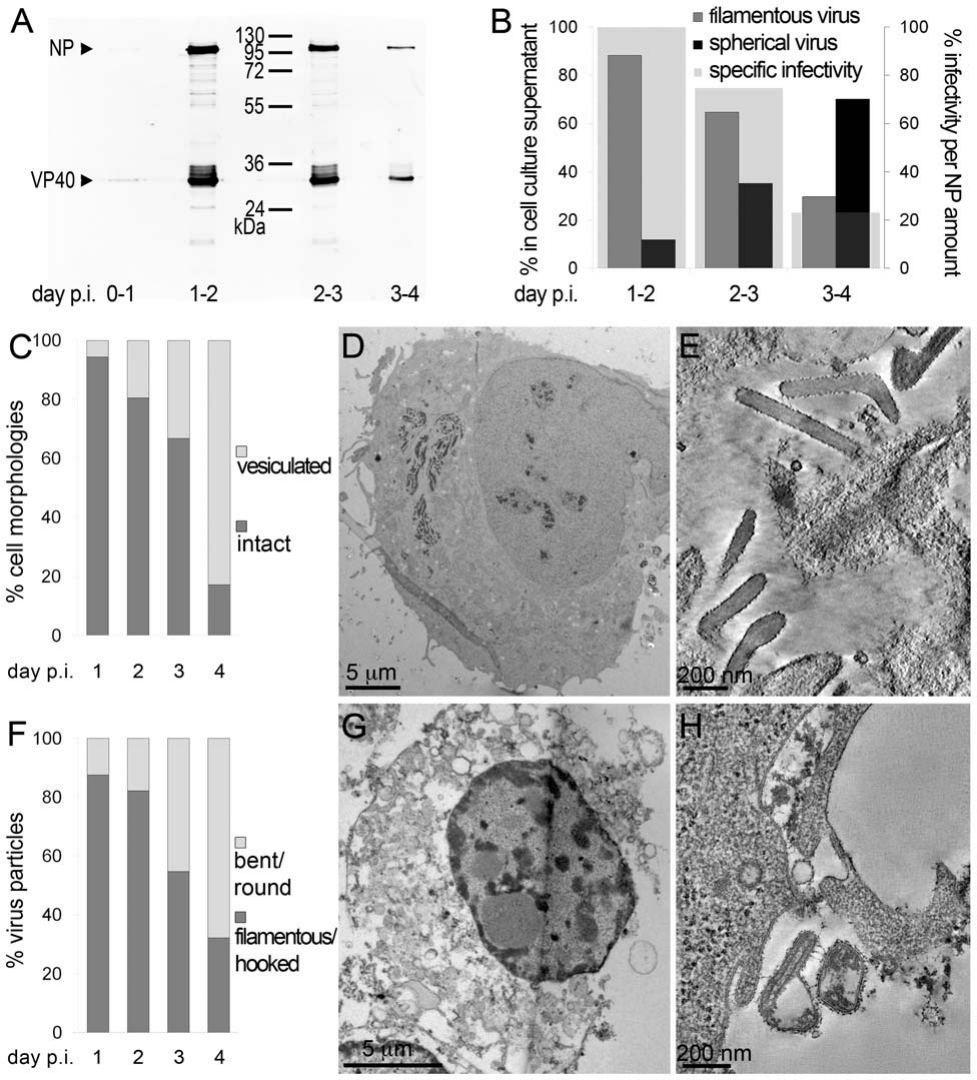
Characterisation of the course of infection in cultured cells. (A) Quantitative Western Blot showing the relative amounts of major viral structural proteins NP and VP40 released into cell culture supernatant during the indicated time periods. (B) Correlation of morphology and specific infectivity of MARV particles released into cell culture supernatant during the indicated time periods. Infectivity in each supernatant was determined in a TCID_50_ assay and normalized for NP amounts in each supernatant to determine the specific infectivity (light grey areas). Relative amounts of filamentous virus (grey bars) and spherical virus (black bars) in each supernatant were determined by EM of thin sections. (C) Quantification of MARV-infected cell morphology over a 4-day infection period. HUH-7 cells were infected, fixed at the indicated time points, embedded in their in situ orientation and cell morphology was determined by EM of thin sections in a systematic random sampling manner. (D) Electron micrograph showing the intact morphology of an infected cell at day 1 p.i.. (E) 6 nm digital slice of a tomogram showing the periphery of an intact cell and the filamentous morphology of the viruses associated with the cell profile. (F) Quantification of morphology of cell-associated MARV particles over a 4-day infection period. Quantification was done as in C. (G) Vesiculated morphology of an infected cell at day 4 p.i.. (H) 8 nm digital slice of a tomogram showing the periphery of a vesiculated cell and the spherical morphology of two viruses and several cell-derived vesicles associated with the cell profile.

To determine the time course of production of infectious MARV during a prolonged infection period, supernatants of HUH-7 cells, infected with MARV under BSL-4 conditions, were collected from day one to four post infection (p.i.) and tested for viral protein content, specific infectivity and virion morphology. The amounts of viral NP and VP40 released from the cells were measured by quantitative immunoblotting. This showed that viral protein release peaked between day one and two p.i. (Figure 1A). The TCID_50_ of each supernatant was determined and normalized to the amount of released NP to estimate the specific infectivity per virus (Figure 1B, grey areas). Specific infectivity was also at a maximum between day one and two p.i.. Virus morphology in the supernatants was monitored by EM and revealed that at the peak of viral protein production and specific infectivity, 80% of the virus particles displayed the characteristic filamentous morphology of the filoviruses, with the remainder appearing bent or round (Figure 1B). Later in infection, the specific infectivity was lower and correlated with fewer filamentous particles and more round or bent particles (Figure 1B), suggesting that infectivity can predominantly be attributed to filamentous virus. There was also an increase in the release of cellular vesicular material over time (Table 1).

**Table 1:** Correlation of virus and cell morphology during the time course of MARV infection.

**Particle shape in cell culture supernatant replaced after each day (** **Figure 1B** **)**					
**virus morphology during time period**	filamentous virus	spherical virus		vesicular material (contains no NC)	total
day 1–2	224	30		2	256
day 2–3	125	68		12	205
day 3–4	51	120		92	263
**Morphology of infected cells and cell-associated virus (** **Figure 1C,F** **)**					
**virus morphology at time point**	filamentous virus		spherical virus		total
day 1	148		21		169
day 2	60		13		73
day 3	37		29		64
day 4	27		57		84
**cell morphology at time point**	intact cell profiles		vesiculated cell profiles		total
day 1	34		2		36
day 2	33		8		41
day 3	20		10		30
day 4	10		48		58

Quantification of virus morphology in cell culture supernatants was done by examination of thawed cryosections of pelleted virus in a systematic random manner. Note that filamentous viruses are predominantly released at day 1–2 p.i., while spherical viruses are predominantly released at day 3–4 p.i.. Cell morphology and morphology of cell-associated virus was assessed by examination of plastic-embedded thin sections of MARV-infected cells, embedded in their in situ orientation, in a systematic random sampling manner. Note the decrease in amount of filamentous virus and of intact cell profiles from day one to day four p.i., and the increasing amount of spherical virus and vesiculated cell profiles over the same time course. For further details see Results section.

Quantitative EM analysis of infected adherent cells that were fixed and embedded in situ revealed that cell morphology also changed over time. At day 1 p.i. 95% of the observed cells appeared intact and displayed filopodia-like PM protrusions (Figure 1C, D). The cytoplasm often contained densely stained viral inclusions (Figure 1D) and virions were readily seen in the immediate periphery of cell profiles; they were easily identified by means of their electron-dense stain and a rod-shaped NC and were frequently found ‘trapped’ between or underneath adherent cells (Figure 1E). Over 90% of all observed virions around the cells were filamentous or hooked (Figure 1F). In contrast, when the monolayer was fixed at 4 days p.i., only 17% of the cell profiles were intact and 83% appeared vesiculated and resembled apoptotic cells (Figure 1C, G). Most of the virus particles around such vesiculated cells were bent or round (Figure 1F, H).

The above characterisation indicates that production of fully infectious, filamentous virus is highest at 1–2 days p.i., when the cells still have intact membrane profiles. This was therefore selected as an appropriate time point for studying assembly and budding of filamentous particles.

### 3D analysis of the steps of MARV assembly, budding and release

**Figure 2:**
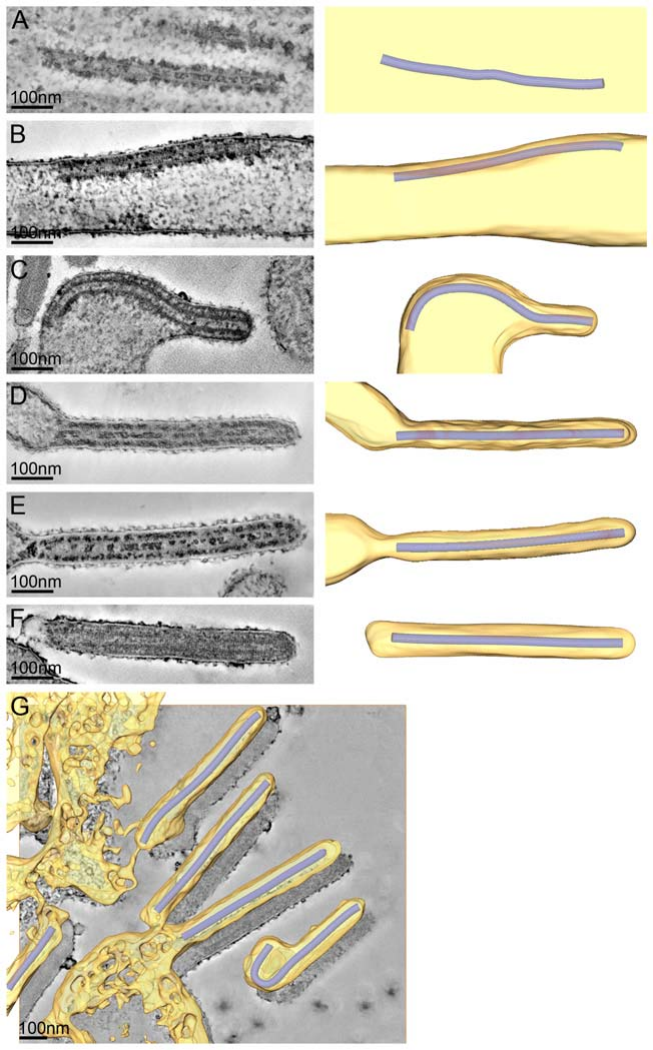
The different steps of MARV budding and release. (A-F) Left panels show digital slices of tomograms of MARV-infected cells at 1 day p.i.. Right panels are the corresponding schematic 3D representations of the viral NC (blue) and the cytoplasm and surrounding membrane (yellow). The different steps of MARV budding and release are (A) Intracellular NC in the cytoplasm. (B) Intracellular NC associated on one side along its whole length with the PM of a filopodia-like membrane protrusion. (C) Viral NC with its tip inserted in a budding structure and associated with the PM along its whole length on one side. (D, E) Viral NCs completely inserted into filamentous budding structures. In (E), a bud neck-like membrane shape is seen at the rear of the budding structure. (F) Filamentous virus with a membrane distortion at one end. Note that all NCs have the same length. Different contrast of NCs in individual images results from differences in electron-dense stain access during sample preparation. (G) 8 nm digital slice of a tomogram of a MARV-infected cell at 1 day p.i.. Coloured overlay is the surface representation of viral NC (blue) and 3D surface rendering of surrounding membrane and cytoplasm (yellow). It shows a filamentous budding structure with the viral NC fully inserted into the budding site and several released virions. 3D surface representation is shifted relative to the tomogram slice for improved visualisation. See also Video S1.

To study the assembly and budding of MARV we performed ET on MARV-infected cells. ET allows the different steps of assembly and budding to be visualized in 3D and the dimensions of viral structures to be measured and analysed. Infected cell monolayers were fixed at 1 day p.i., removed from the BSL-4 facility, processed for EM and cut into 300 nm thick sections in their in situ orientation. Dual-axis tilt series of the sections were acquired in the electron microscope, as described in Materials and Methods, and 3D reconstructions were computationally generated. Viral NCs could be readily identified in 3D reconstructions. They were found around viral inclusions, within the cytoplasm, associated with the PM, incorporated into budding viruses, and in released virus particles (Figure 2). We therefore used the NC itself as a convenient marker for identifying virus assembly intermediates.

The 3D data allowed us to measure the length of complete viral NCs even when they were bent or tilted with respect to the sectioning plane. In the periphery of viral inclusions in the cytoplasm individual NCs could be found. They appeared as rod-shaped, striated structures that were more densely stained than the cytoplasm and were on average 711 nm long (Figure 2A, Table 2). NCs were frequently seen at the PM or in filopodia-like membrane protrusions, where they were associated with the membrane along their whole length. The length of PM-associated NCs was 707 nm (Figure 2B, Table 2), the same as that of cytoplasmic NCs. Viral budding structures localized predominantly to filopodia-like protrusions of infected cells, in agreement with previous data [23]. They appeared as filamentous finger-like extensions emerging either from the tip or the sides of filopodia-like protrusions (Figure 2C-E). Each bud accommodated a rod-shaped NC that was surrounded by densely stained material and had the same length as intracellular and PM-associated NCs (Table 2). Of the budding structures reconstructed in 3D, 13% appeared as extrusion intermediates (Table 2). These structures contained a full length NC (729 nm) that was only partially extruded (Figure 2C): one end of the NC was tightly wrapped on all sides by the PM, whereas the other was attached to the PM along one side (Figure 2C). The majority (87%) of budding structures had the NC completely inserted into the finger-like membrane extension (Figure 2D, E). Scission at the base of these fully extruded buds would lead to the release of filamentous virions (Figure 2F and Video S1).

**Table 2:** 3D analysis of virus shape and NC length at different steps of filamentous MARV budding.

3D measurements in tomograms			
**filamentous virus dimensions**			
length	789.3 nm	±61.0 nm	(n = 20)*
diameter	88.0 nm	±11.8 nm	(n = 21)
**shape of virus ends**			
	19/21 viruses have one distorted end and one hemisphere		
	2/21 viruses have two hemispheres		
**nucleocapsid length**			
intracellular	711.1 nm	±68.3 nm	(n = 7)
plasma membrane associated	707.7 nm	±126.8 nm	(n = 17)
in partially extruded buds	728.5 nm	±45.7 nm	(n = 3)
in fully extruded buds	736.5 nm	±52.7 nm	(n = 20)
in released filamentous virus	735.1 nm	±55.0 nm	(n = 18)**
in released spherical virus	734.0 nm	±108.0 nm	(n = 19)

3D measurements of filamentous virus dimensions and identification of filamentous virus end shapes were carried out in 3D tomographic reconstructions of MARV-infected HUH-7 cells at 1 day p.i.. NC length measurements were taken in 3D on NCs completely included in tomographic reconstructions of MARV-infected HUH-7 cells at 1 and 4 days p.i.. All NCs in the cytoplasm, in budding structures, in filamentous viruses and in spherical particles have the same average length.

*excluding one virus outlier of double length (1518 nm).

**excluding one nucleocapsid outlier of double length (1472 nm), and two particles where the NC ends could not be reliably defined.

Most released viruses in the periphery of infected cells appeared as straight filaments in 3D reconstructions (Figure 2F). Some filamentous viruses displayed one bent or buckled end giving rise to hooked or six-shaped particles (Figure 2G and Video S1). Filamentous viruses were on average 789 nm long and had a diameter of 88 nm (Table 2), in agreement with previous studies [20], [24]. All virions had a membrane envelope and contained a rod-shaped NC that displayed the regular striated pattern previously described [12], [20]. The volume between membrane envelope and NC was filled with a densely stained material, most likely the VP40 matrix protein, which appeared to link the envelope to the NC on all sides and along the whole length (Figure 2F). 3D analysis revealed that NCs in all released virions had the same length, on average 735 nm, and appeared bent or kinked when particles were hooked or six-shaped (Figure 2G, Table 2). These length averages exclude a single outlier, which was a double-length filamentous virus with a double-length NC (Table 2). The presence of NCs of the same length in the cytoplasm, at the PM, in filopodia, in all budding structures and in filamentous viruses suggests that the NC assembles into a helix of a defined and final length prior to being transported to the PM.

**Figure 3:**
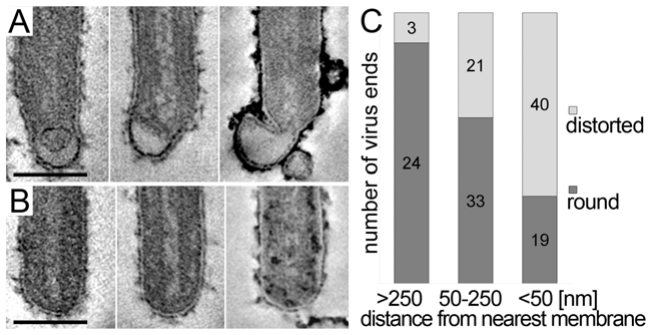
Membrane distortions are found at the rear end of filamentous MARV particles. MARV infected cells were embedded and sectioned in their in situ orientation and the morphology of filamentous viruses was examined in electron tomograms of 300 nm thick sections. (A, B) Digital slices of tomograms showing typical examples of (A) distorted ends and (B) intact, round ends of filamentous viruses. Scale bars 100 nm. (C) Quantification of the distance of distorted and round virus ends from the nearest cellular membrane. Distances were measured in 3D tomograms and assigned to one of three distance classes >250 nm, 50–250 nm, <50 nm. At a distance <50 nm from the nearest cell membrane, most virus ends are distorted. At a distance >250 nm away from the nearest cell membrane the majority of virus ends is intact and round.

The 3D data also allowed us to closely examine both ends of reconstructed filamentous virions. This revealed morphological differences between the two virus ends: 19 out of 21 3D-reconstructed filamentous virions displayed a membrane bulge or hook at one end (Figure 3A), while the membrane formed a round, intact hemisphere at the opposite tip (Figure 3B and Table 2). Only 2 out of 21 filamentous viruses had two round, intact tips. Virions with membrane distortions at both ends were never observed. Although we cannot exclude that such membrane distortions are due to chemical fixation, they suggest that a local instability of the viral membrane is specifically induced on one virus end. Thus, we made use of the fact that cells and extracellular viruses were fixed and examined in their in situ orientation. Measurement of the average distance of intact and distorted virus ends from the nearest cellular membrane revealed that intact virus ends were predominantly found at distances >250 nm away from a cellular membrane, whereas distorted virus ends were more frequently localized within 50 nm distance (Figure 3C). This suggests that the membrane distortions are found at the rear end of filamentous viruses where scission took place (also shown in Figure 2G and Video S1).

### Virus release during late rounds of infection

**Figure 4:**
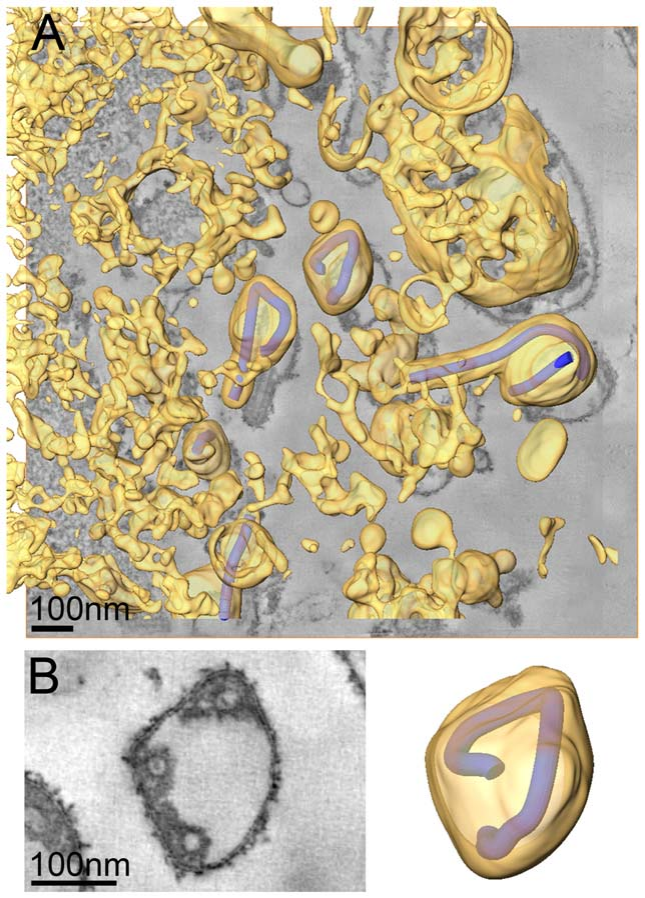
Release and spherical morphology of MARV particles after prolonged infection. (A) 8 nm digital slice of a tomogram taken from a MARV-infected cell at 4 days p.i.. Coloured overlay is the surface representation of viral NCs (blue) and 3D surface rendering of cytoplasm and membranes (yellow). Several spherical viruses and a six-shaped particle near the surface of a vesiculated cell and surrounded by cell-derived vesicles and debris are shown. 3D surface representation is shifted relative to the tomogram slice for improved visualisation. See also Video S2. (B) Left panel is a digital slice of a tomogram taken from a MARV-infected cell at 4 days p.i. showing the round shape of a virus and three cross sections of the viral NC. Right panel is the corresponding schematic 3D representation of the viral NC (blue) and the viral membrane (yellow). Note that the 3D reconstruction reveals that the viral NC is kinked but continuous.

We also carried out ET of infected cells fixed at 4 days p.i.. At this time point the majority of cells exhibited heavily vesiculated membranes and produced rounded viruses of lower infectivity. The 3D analysis revealed that most viruses were roughly spherical in shape. They were generally found in close proximity to convoluted, vesiculating areas of the PM (figure 4A) and often surrounded by large numbers of cell-derived vesicles (Figure 4A and Video S2). This suggests that release of spherical viruses occurs rapidly and simultaneously with the shedding of other cell-derived vesicles. NCs within spherical virions were bent or kinked but never broken or segmented, as might be suggested by 2D EM of thin sections (see, for example the 2D and 3D views of the spherical particle shown in Figure 4B). None of the virus particles were toroidal, as has previously been suggested [39]. Interestingly, kinked NCs in spherical viruses had a total length of 734 nm, the same length as NCs in filamentous virions (Figure 4B, Table 2) and they were associated along one side with the viral membrane (Figure 4B). These findings demonstrate that preassembled full-length NCs are packaged into each virion, irrespective of virus shape.

## Discussion

The analysis of MARV-infected cells by ET allowed us to take 3D measurements of NCs and revealed that NCs have uniform length at all stages in the virus assembly and budding process described here, from intracellular NCs to released virus particles. This suggests that the length of the complete viral genome and the number of nucleoproteins required to encapsidate the genome dictate the length of the intracellular NC prior to its transport to the PM.

**Figure 5:**
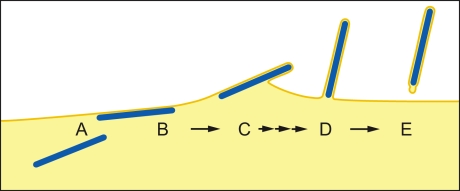
Steps of filamentous virus budding and release. (A) An individual viral NC in the cytoplasm of an infected cell. (B) The viral NC is delivered in full-length form to the PM, with which it associates along one side for its entire length. (C) Slow initiation of NC envelopment at one end of the NC at the PM. (D) Envelopment proceeds in a fast process along the length of the NC until the nascent virion protrudes from the membrane, remaining attached at only one end. (E) Scission of the virus particle from the PM leaves a local membrane instability at the rear end of the virus particle. For details see Discussion. The model is based on 3D tomographic reconstructions of MARV-infected cell sections; Membrane and cytoplasm are depicted in yellow, NCs are blue. Single and triple arrows depict slow and fast processes, respectively.

Once the NC has reached the PM, two contrasting mechanisms have been proposed for filovirus budding. In the “submarine-like” model, budding proceeds via lateral association of the NC with the membrane, followed by horizontal budding. In the “rocket-like” model, the NC is extruded vertically from the PM. The analysis of budding MARV particles presented here allows the sequence of steps resulting in filamentous virus budding and release to be described in 3D. Viral NCs are assembled in the cytoplasm (Figure 5A) and delivered in their full-length form to the PM, with which they associate laterally, along one side, for their entire length (Figure 5B). Envelopment of the PM-associated NC is initiated at one end, and proceeds along the length of the NC (Figure 5C) until the nascent virion protrudes from the membrane, remaining attached at only one end (Figure 5D). Only very few particles are seen which appear to be partly extruded, whereas larger numbers of NCs are either associated with the PM prior to extrusion or are fully extruded and protruding from the PM. This strongly suggests that extrusion is a rapid process in comparison with its initiation or the subsequent membrane scission event.

Scission of the filamentous virus particle from the PM then takes place with a bud-neck shape which can have a circular cross-section. This is the same shape as the bud-neck which would be present in the budding of spherical enveloped virions, or in the budding of vesicles into a multi-vesicular body [40]. Release of a horizontally budding particle would require scission of a membrane neck with a non-circular cross-section. Our observations indicate that no such unusual scission mechanism needs to be proposed.

Scission of the protruding bud leaves local membrane instability at the rear end of the virus particle (Figure 5E), which may be exaggerated during sample preparation. In contrast, the front end of the filamentous particle is a well-defined hemisphere. The difference between the two ends could reflect a destabilization at the rear end of the particle induced by scission. Alternatively, the hemispherical front end of the particle may be stabilized by a structure involved in initiating envelopment, similar to the front end of bullet-shaped rhabdovirus particles [41], [42].

The budding of filamentous MARV therefore proceeds via an NC that is laterally associated with the PM, and a vertically protruding bud. Any changes in the rates of individual steps in the budding process could dramatically alter the appearance of the budding structures in EM. For example, if the rate of scission were significantly increased, or the rate at which extrusion is initiated were dramatically decreased, then large numbers of NCs might be expected to collect horizontally under the PM, giving the appearance of a predominant “submarine” mode of budding [36]. If the rate of initiation of extrusion were significantly increased, or the rate of scission decreased (for example by inhibiting recruitment or function of the cellular endosomal protein sorting system), then large numbers of protruding buds would accumulate, giving the appearance of a predominant “rocket-like” mode of budding. We suggest the contrasting appearance of Ebola virus and MARV infected cells in EM does not reflect different budding mechanisms, but rather different rates for the individual steps of the process presented here. These rates are likely to be both virus and cell type dependent.

The sequence of steps in budding described here results in a very different mechanistic understanding of filovirus budding. Firstly, the proposed two budding modes, one vertical and one horizontal, can be reconciled as representing different snapshots of a single budding mechanism. Secondly, rather than filoviruses adopting a unique horizontal mode of budding not seen in other systems, the budding process can now be placed within the established framework of other cellular budding events. In the new model, budding is initiated by wrapping of one end of the NC with a hemispherical membrane, and completed by scission at a bud-neck with a classic round cross-section. These are the membrane shapes present during budding and scission events of cellular vesicles and spherical viruses. Thirdly, comparison of our data with published electron micrographs of filamentous virus budding structures suggests that other viruses may also follow the sequence of steps described here, and that rather than being unique, filovirus budding belongs within a more general budding mechanism also adopted by other rod-shaped viruses. For example, budding VSV, a rhabdovirus, can be found protruding perpendicular to cell membranes in infected cells, but the NC can also be seen underneath the cell membrane, with which it associates laterally along one side [35]. The rounded end of the virus, easily distinguished in VSV, seems to associate more tightly with the membrane [43], and buds first. After budding, under certain preparation conditions, the rear of the virus is seen to show small membrane blebs or instabilities [41] similar in appearance to those described here for MARV. These striking observations are consistent with VSV following the same sequence of budding steps as MARV. A general assembly and budding mechanism for filoviruses and rhabdoviruses might represent a target for future antiviral drugs.

After a prolonged period of infection, the released MARV particles have lower specific infectivity and the majority is roughly spherical in form. Like the filamentous virions, the spherical particles contain a single full-length NC, which is kinked in a number of places, but not broken, suggesting that these particles still contain a full-length viral genome. The NC is not tightly wrapped on all sides as it is in filamentous virions, but is associated with the membrane along one side for its entire length. This lateral association is also seen for intracellular NCs prior to extrusion from the PM. Since formation of spherical particles is paralleled by convolution of the PM and shedding of cellular vesicular material into the supernatant, we propose that spherical viruses are released by large-scale membrane instability at sites of budding, leading to vesiculation of viruses prior to extrusion. In this model, kinking of the NC might be induced by the forces which lead to invagination and vesiculation of the PM, though other factors could contribute, such as lack of a viral or cellular factor which rigidifies the NC. The interplay between the budding process and the dynamics or composition of the cellular membrane, as well as possible changes in the relative rates of the different steps of the budding process in different virus strains [44], mutants [45], cell types [46] or stages of infection, may also contribute to the variable morphology observed in other viruses.

In summary, our observations reconcile the contrasting models of filovirus budding and allow us to describe the sequence of events taking place during budding and release of MARV virus. We propose that this represents a general sequence of events also followed by other filamentous and rod-shaped viruses. Furthermore, we demonstrate that virus shape is determined both by viral and cellular factors. The model for filamentous virus budding presented here raises a number of new questions. Do elements of the cytoskeleton or other cellular components play a specific role in mediating envelopment or budding of filamentous or spherical virus particles? Is one end of the PM-associated NC specifically able to initiate the extrusion of the filament, as in rhabdoviruses, or is the directionality of the extrusion process random? What is the organisation of the NC and VP40 matrix layers in the released virion? What kind of arrangement of interactions between NC and VP40 occurs in the wrapping process during bud extrusion? These and other questions must be addressed in future structural studies, and may have wider implications for the budding and assembly of filamentous viruses.

## Materials and Methods

### Cells and viruses

The HUH-7 human hepatoma cell line and Vero cells were maintained in Dulbecco's modified Eagle medium (DMEM) supplemented with 10% fetal calf serum, l-glutamine and penicillin–streptomycin at 37°C under 5% CO_2_. All work with infectious MARV was performed under BSL-4 conditions at the Institute of Virology in Marburg. The MARV Leiden strain, isolated in 2008 in Leiden, the Netherlands [47], was propagated in Vero E6 cells and purified as described previously [48]. HUH-7 cells were infected with MARV with a multiplicity of infection of approximately 1 plaque-forming unit per cell for one to four days. At the indicated time points, cell culture supernatants were collected and used for viral infectivity assays (see below), or viruses in the supernatants were purified by centrifugation over a sucrose cushion and fixed for 48 hours (h) with 4% paraformaldehyde in PBS for analysis of particle morphology (see below). For EM and ET of infected cells, HUH-7 cells were grown on carbon-coated sapphire disks, infected as above and cell monolayers were fixed at the indicated time points in the cell culture dish with 4% paraformaldehyde/0.1% glutaraldehyde in 0.1M PHEM buffer (60 mM PIPES, 25 mM HEPES, 2 mM MgCl_2_, 10 mM EGTA), pH 6.9 for 30 min, after which the fixative was replaced with 4% paraformaldehyde in 0.1M PHEM. Fixed cells were removed from the BSL-4 lab after 48 h of inactivation.

### Viral infectivity assay

Infectivity of MARV particles released into the supernatant of cells 1 to 4 days p.i. was assayed by a 50% tissue culture infective dose (TCID_50_) assay: Vero cells were grown in 96-well plates to 30 to 40% confluence. Cells were inoculated in quadruplicate with 10-fold serial dilutions of supernatants of HUH-7 cells infected with the MARV Leiden strain for one to four days as described above. The assays were evaluated at 10 days p.i.. TCID_50_ values were calculated using the Spearman-Karber method [49]. Equal volumes of each supernatant were separated by SDS-PAGE followed by quantitative immunoblotting on a LiCor Odyssey system using a mouse monoclonal anti-NP antibody, and secondary antibodies, protocols and software (Odyssey version 2.0) provided by the manufacturer. TCID_50_ values were normalized to NP levels detected in each supernatant, and the TCID_50_ value in the supernatant collected at day 2 p.i. was set to 100%.

### Sample preparation for electron microscopy

For quantification of virus morphology at different time points p.i., fixed cell culture supernatants were purified by centrifugation over a sucrose cushion, pelleted for 30 min at 40,000 g, 4°C in a Beckman ultracentrifuge using a TLA-55 rotor. Pellets were embedded in 12% gelatin and prepared for EM as described previously [50]. 70 nm cryosections were obtained with a Leica EM UC6 microtome, FC6 cryochamber (Leica Microsystems, Wetzlar, Germany) and a diamond knife (Diatome, DiS-Galetzka Weinheim, Germany). Thawed cryosections were counterstained with uranyl acetate as described elsewhere [51]. For EM and ET of infected cells, HUH-7 cells were grown on carbon-coated sapphire disks, infected and fixed as described above. Samples were high-pressure frozen with a BalTec HPM-010 and freeze substituted with 0.1% (w/v) uranyl acetate, 1% osmium tetroxide (w/v) and 5% (v/v) water in glass distilled acetone in a temperature-controlling device (Leica EM AFS I). Cells were kept for 40 h at −90°C and warmed up to 0°C (slope 5°C/h) with an additional 3 h infiltration period at −30°C. Samples were washed three times with glass distilled acetone, infiltrated at room temperature with increasing concentrations of epoxy resin (Glycidether 100, Roth, Karlsruhe, Germany) in acetone over 12 h, and polymerized at 60°C for 48 h. 150 nm and 300 nm sections were obtained with a Leica Ultracut UCT microtome and a diamond knife.

### Electron microscopy and tomography

Thin sections of virus and infected cells were examined on a FEI Morgagni 268 TEM equipped with a 1K side mounted CCD camera (SIS, Muenster, Germany). Quantification of cell morphology and cell-associated virus morphology was carried out on 150 nm resin-embedded sections in a systematic random sampling manner [52]. Starting points for sampling were chosen randomly on each grid, and all grids were examined in the same systematic manner. At least three grid squares per EM grid and three EM grids per time point were sampled. Morphology of viruses from cell culture supernatants was quantified by EM in the same systematic random sampling manner on thawed 70 nm cryosections of virus pellets, purified and embedded as described above. At least 200 particles from three different grids were evaluated per sample. ET was carried out essentially as described elsewhere [53]. Dual axis tilt series from 300 nm sections were recorded on a FEI TECNAI TF30 microscope operated at 300kV (4K FEI Eagle camera; binned pixel size 0.77 nm or 1 nm on the specimen level) over a −60° to 60° tilt range (increment 1°) and at a defocus of −0.2 µm. Tomograms were reconstructed using the IMOD software package (version 3.12.20) [54]. 3D measurements of virus end distance, analysis of virus end morphology and 3D surface renderings were carried out using the AMIRA Visualisation Package (version 5.2.0, Visage Imaging, Berlin, Germany). In total, 68 3D reconstructions from three independent infection experiments were analysed, of which 48 of high image quality were used for detailed analysis and measurements. NCs in different samples displayed variable contrast, which resulted from differential access of electron-dense stain to individual cells and different subcellular regions during sample preparation. NC representations in Figures 2 and 4 and Video S1 and Video S2 were generated and displayed using the AMIRA EM package [55] and MatLab (version 7.4.287).

## Supporting Information

Video S1 Release of filamentous Marburg virus particles at one day post infection. Animation through a z-series of 2 nm digital slices of a double axis tomogram, reconstructed from a tilt series taken of a ∼300 nm thick section of a MARV-infected HUH-7 cell at one day post infection. Coloured overlay shows a 3D surface model of membranes and cytoplasm (yellow) and representations of the viral NCs (blue). A filamentous viral budding structure with the NC completely inserted into the budding site as well as several released filamentous viruses are seen.
(3.46 MB MOV)

Video S2 Release of spherical Marburg virus particles at four days post infection. Animation through a z-series of 2 nm digital slices of a double axis tomogram, reconstructed from a tilt series taken of a ∼300 nm thick section of a MARV-infected HUH-7 cell at four days post infection. Coloured overlay shows a 3D surface model of membranes and cytoplasm (yellow) and representations of the viral NCs (blue). Several spherical and six-shaped virus particles are seen near the cell surface and surrounded by cell-derived vesicles and debris.
(8.67 MB MOV)
